# Transplant management in Brazil: a temporal analysis of financial investments and procedures

**DOI:** 10.1590/1980-220X-REEUSP-2024-0039en

**Published:** 2024-08-23

**Authors:** Mercy da Costa Souza, Marcos Antonio Ferreira, Carolina Mariano Pompeo, Felipe Machado Mota, Elenir Rose Jardim Cury

**Affiliations:** 1 Universidade Federal de Mato Grosso do Sul, Programa de Pós-Graduação em Saúde e Desenvolvimento na Região Centro-Oeste. Campo Grande, MS, Brazil.; 2 Universidade Federal de Mato Grosso do Sul, Programa de Pós-Graduação em Enfermagem do Instituto Integrado de Saúde. Campo Grande, MS, Brazil.

**Keywords:** Organ Transplantation, Tissue and Organ Procurement, Costs and Cost Analysis, Health Services Accessibility, Epidemiology, Trasplante de Órganos, Obtención de Tejidos y Órganos, Costos y Análisis de Costo, Accesibilidad a los Servicios de Salud, Epidemiología

## Abstract

**Objective:**

To analyze public management actions regarding organ, cell, and tissue transplant procedures and their financial investments in Brazil.

**Method:**

Mixed (time and place) ecological study, carried out based on data from the Hospital Information System of the Brazilian Public Health System (SUS) Information Technology Department and the National Transplant System, from 2001 to 2023. Temporal trend analyses, descriptive and inferential statistics were performed.

**Results:**

Organ, cell, and tissue transplants are concentrated in the Southeast region of the country, with increased costs there. The Northeast and South regions of Brazil have the longest waiting list, with an increasing trend (R^2^ = 0.96), associated with a decreasing trend in the number of transplants (R^2^ = 0.97).

**Conclusion:**

The difference in the total number of transplants and procedures performed among the Brazilian regions represents the need for organization and investments with strategies aimed at training professionals and raising awareness among the population.

## RESUMEN

**Objetivo:**

Analizar acciones de gestión pública en materia de inversiones financieras y procedimientos de trasplante de órganos, células y tejidos en Brasil.

**Método:**

Estudio ecológico mixto (tiempo y lugar), realizado con datos del Sistema de Información Hospitalaria del Sistema Público Brasileño de Salud (SUS), del Departamento de Informática del SUS y del Sistema Nacional de Trasplantes, de 2001 a 2023. Se realizaron análisis de tendencias temporales, estadística descriptiva e inferencial.

**Resultados:**

Los trasplantes de órganos, células y tejidos se concentran en la región Sudeste del país, donde los costos aumentan. Las regiones Nordeste y Sur de Brasil tienen la lista de espera más larga, con tendencia creciente (R^2^ = 0,96), asociada a una tendencia decreciente en el número de trasplantes (R^2^ = 0,97).

**Conclusión:**

La diferencia en el número total de trasplantes y procedimientos realizados entre regiones de Brasil representa la necesidad de organización e inversiones con estrategias orientadas a la formación de profesionales y la sensibilización de la población.

## INTRODUCTION

The demand for transplants is growing considerably all over the world due to the increase in chronic and degenerative non-communicable diseases, such as diabetes, hypertension and cancer, among other causes. This replacement therapy aims to provide clinical improvements for individuals whose health conditions do not respond adequately to conventional or less invasive treatments^(1–3)^.

The World Health Organization reports that approximately 130,000 solid organ (SO) transplants are performed per year, which represents only 10% of the global need. However, the estimate is that in Brazil this rate could reach up to 30%, due to its large public program, the National Transplant System (*SNT*)^(4)^.

For the donation and transplantation process to be carried out in Brazil, the *SNT*, under the guidelines of the Ministry of Health (MS), operates in accordance with Law No. 9.434/1997, which provides fundamental support and determines that the *SNT* regulates, coordinates and supervise the entire network of activities of the public transplant system in the national territory^(5,6)^.

Transplant financing and management varies around the world^(7,8)^. While Central American countries deal with fragmented and heterogeneous healthcare systems, with partial government funding^(9,10)^, the Brazilian Public Health System (*SUS*) is internationally recognized for providing comprehensive and free pre- and post-transplant care, with more than 90% of procedures paid for by the public authorities^(3,5,6)^.

Although *SUS* finances procedures related to transplants and the Brazilian public program is internationally recognized^(11)^, the number of replacement therapies carried out still does not meet the population’s demand. In December 2023, only 15% of patients on the transplant waiting list were treated, resulting in 59,958 individuals who, at the end of the year, were still waiting for transplantation^(12)^.

This high number of people on the waiting list varies within Brazilian regions and represents each one’s different investment and management capabilities. Due to Brazil’s continental characteristics, its five regions – North, Northeast, Southeast, Central-West and South – present distinct geographic, demographic, and socioeconomic particularities^(11)^. These differences result in varying levels of investment in health and directly influence the logistics, infrastructure, organ procurement, and transplantation capacity of each location.

Transplantation is a highly effective treatment for advanced organ failure and is often the only therapeutic option indicated to prolong an individual’s life^(1)^. As the number of chronic non-communicable diseases with potentially organ-damaging effects continues to grow, the demand for this service will inevitably increase^(3)^.

Therefore, if there is no management with efficient investments, according to the particularities of each region, the single list, which already has a large number of patients, will cover more and more people who need this procedure^(1)^. Therefore, it is essential to determine the investment profile and procedures carried out in each region to understand how government management policies can impact the efficiency of transplant services in the country.

Additionally, Brazilian literature presents a significant gap in evidence regarding the systemic, infrastructural, and geographic challenges faced by the *SUS*, which may compromise individuals’ ability to access transplant services and obtain high-quality treatments^(3)^. Given theses conditions, this study has the objective of analyzing public management actions regarding organ, cell, and tissue transplant procedures and their financial investments in Brazil.

## METHOD

### Design of Study

Epidemiological study, with a mixed ecological design (time and place), carried out using secondary data from the *SUS* Hospital Information System (SIH/SUS) of the *SUS* Information Technology Department (DATASUS) and statistical reports from the *SNT*.

### Population, Local and Selection Criteria

The mixed ecological study was carried out with data from population aggregates from the five Brazilian regions, Central-West, North, Northeast, Southeast and South, which were obtained from two distinct information sources: the *SIH/SUS* and the *SNT* statistical reports. The definition of the study population was carried out based on the individual characteristics of each base.

For *SIH/SUS* data, all records of hospital admissions paid for by the *SUS* from 2008 to 2023 were included. Regarding data from the *SNT* statistical reports, the population used was derived from transplants and organs, tissues, and cells donations carried out between 2001 and 2021, as well as the waiting list for the period between 2008 and 2021. The time frame for both databases was defined according to data availability during the collection period.

The selection of data obtained through *SIH/SUS* was defined by applying the region/federation unit filter, year of processing (2008 to 2023), procedures (number of hospitalizations, costs and days of hospitalization related to transplants), and subgroup of procedures performed for transplantation. For the *SNT* reports, all data made available in the document were included.

The use of two distinct sources of information to define the population was intended to cover and detail the object of study, since while the *SIH/SUS* provides more specific information such as number of hospitalization, days of hospitalization, value and procedures related to transplants, the *SNT* reports provide numbers referring to the number of donors, patients on the waiting list, percentage of family denial and, finally, the number of transplants carried out.

### Data Collection

Data collection was carried out from July 1st to 31st, 2023. All information related to organ, tissue and cell transplantation from January 2008 to March 2023 was extracted via the internet from *SIH/SUS* using TabNet Win32 3.0. The data collected referred to the number of hospitalizations, costs, and days of hospitalization for transplant-related procedures.

The variables collected were: total hospitalizations for organ, tissue and cell transplantation, total cost of the transplant, average days of hospitalization for transplant procedures. In addition, data relating to subgroups of procedures performed for transplantation were included: collection and examinations for the purpose of donating organs, tissues and cells and for transplants; actions related to the donation of organs, cells and tissues for transplantation; and transplantation of organs, tissues and cells.

In a second stage, data from *SNT* reports related to transplantation and donation of organs, tissues and cells from 2001 to 2021 and the waiting list from 2008 to 2021 were obtained. The variables collected from the *SNT* are related to the total number of patients on the waiting list for organs and tissues; number of solid organ (SO) and cornea transplants, in absolute number and per million population (pmp); total of potential donors (PD) and actual donors (AD); waiting list for SO and corneas; absolute number and percentage of family refusal and organ donation effectuation. After the collection period, the information was exported to a *Microsoft software Excel* ^®^ spreadsheet for tabulation.

### Data Analysis and Treatment

Data were analyzed individually according to each source adopted, that is, there was no crossing of data between *SIH/SUS* and statistical reports from the *SNT*. Descriptive analyses and inferences were carried out according to the characteristics of each data. Missing data were not considered for inferential analysis.

A temporal trend analysis was carried out to verify the historical behavior of the variables investigated using the simple moving average (SMA) technique calculated in three-year cycles using the formula (SMA = (P1 + P2 + P3)/3). Subsequently, temporal graphs were constructed to represent the total number of patients on the waiting list specifically for SO, as well as for the waiting list for a cornea. Furthermore, the total number of transplants and transplants per pmp, the number of SO and corneal transplants pmp and the total PD and AD were also considered to verify the possible shape of the trend curve to be studied.

After this process, polynomial regression models were applied, so that the model that best suited the curve was the third degree model, also called cubic and represented by the formula (Y = β_0 + β_1 X + β_1 X^2 + β_1X^3). The models were chosen according to the highest coefficient of determination (R^2^). The primary outcomes used in the trend analysis were: the trend of increase or decline over time in the number – absolute and pmp – of transplants and patients on the waiting list and absolute number of potential donors.

To analyze procedures related to transplantation, inferential analysis was performed. Shapiro-Wilk test was applied to check the normality of data distribution. Afterwards, the one-way Anova test was applied to variables with normal distribution and the Kruskal-Wallis test if the assumption of normality has not been met. The Games-Howell and Dunn Post-Hoc tests were used to evaluate the statistical difference found among regions.

In inferential group analyses, the following primary outcomes were evaluated: the difference – in absolute number – of effective donors; family interviews and denials; percentage of completion; cornea and SO waiting list; corneal and SO transplants in the Brazilian regions. All data was taken from *SNT* reports.

Regarding group analysis of data taken from *SIH/SUS*, the primary outcomes studied were: the number of hospitalizations; cost in reais, and days of stay for collection and examinations for the purpose of donating organs, tissues, and cells, and transplants; actions related to the donation of organs and tissues for transplantation; and transplantation of organs, tissues and cells among the Brazilian regions.

To verify the correlation between the variables, tests were carried out: Pearson for parametric variables and Spearman for non-parametric ones. For all tests, a significance level of 0.05 was used.

### Ethical Aspects

As data were of public domain and non-nominal, there was no need for prior submission and approval by a Human Research Ethics Committee.

## RESULTS

Regarding the transplant waiting list obtained through *SNT* data from 2008 to 2021, 463,637 patients waited for a solid organ and 213,823 for a cornea transplant at some point in this period, with an average of 33,116 (SD = 4,328.88) and 15,273.07 (SD = 5,244.99), respectively. The years with the highest number of people waiting for an organ were 2008 and 2009, with 64,275 and 63,866 each.

**Figure 1:**
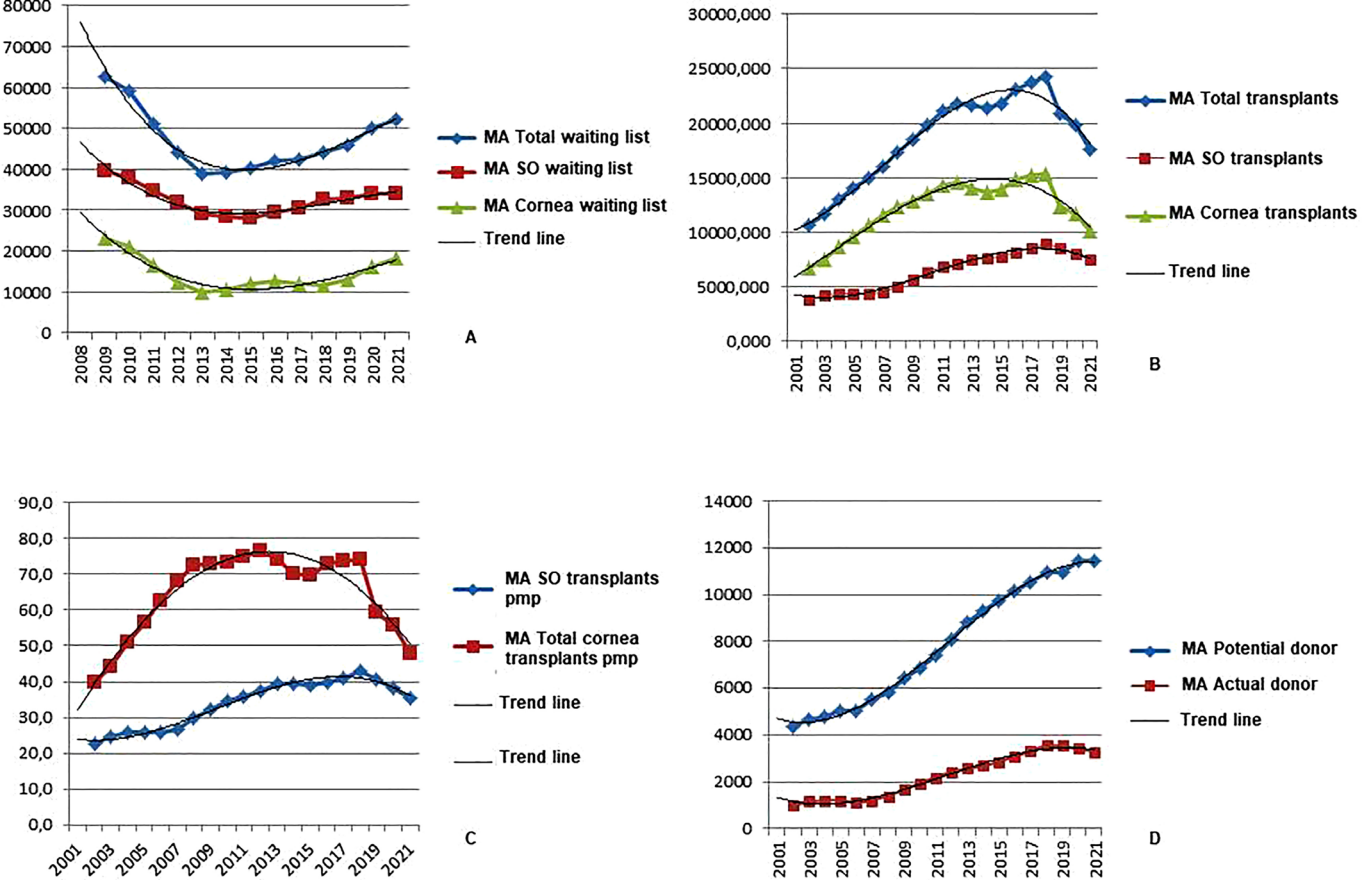
Time trend curve and moving average of the waiting list for transplants from 2008 to 2021, number of transplants and number of transplants per million population (solid organs and corneas), number of potential donors and actual donors from 2001 to 2021 – Campo Grande, MS, Brazil, 2024.

**Legend:** MA = Moving Average; SO = Solid organs; pmp = Per million population.

**Note:** (A) Moving average and trend curve of the waiting list for solid organs and corneas (R^2^ = 0.96); the waiting list for solid organs (R^2^ = 0.94); the cornea waiting list (R^2^ = 0.91); (B) Moving average and trend curve for the number of solid organ and cornea transplants (R^2^ = 0.97); the number of solid organ transplants (R^2^ = 0.94); the number of corneal transplants (R^2^ = 0.98); (C) Moving average and trend curve of the rate of solid organ transplants per million population (R^2^ = 0.93); the rate of corneal transplants per million population (R^2^ = 0.98); (D) Moving average and trend curve of the number of potential donors (R^2^ = 0.99) and actual donors (R^2^ = 0.99).

The average PD over the period was 385.27 (SD = 190.53) and the AD found was 100.11 (SD = 78.45) pmp. An average of 1,341.67 (SD = 928.86) interviews were carried out with the PD’s family, with an average of 504.88 (SD = 345.27) refusals to donate organs and tissues. Figure 1 presents the moving average and temporal trend of data related to transplantation between 2001 and 2021.

**Figure 2:**
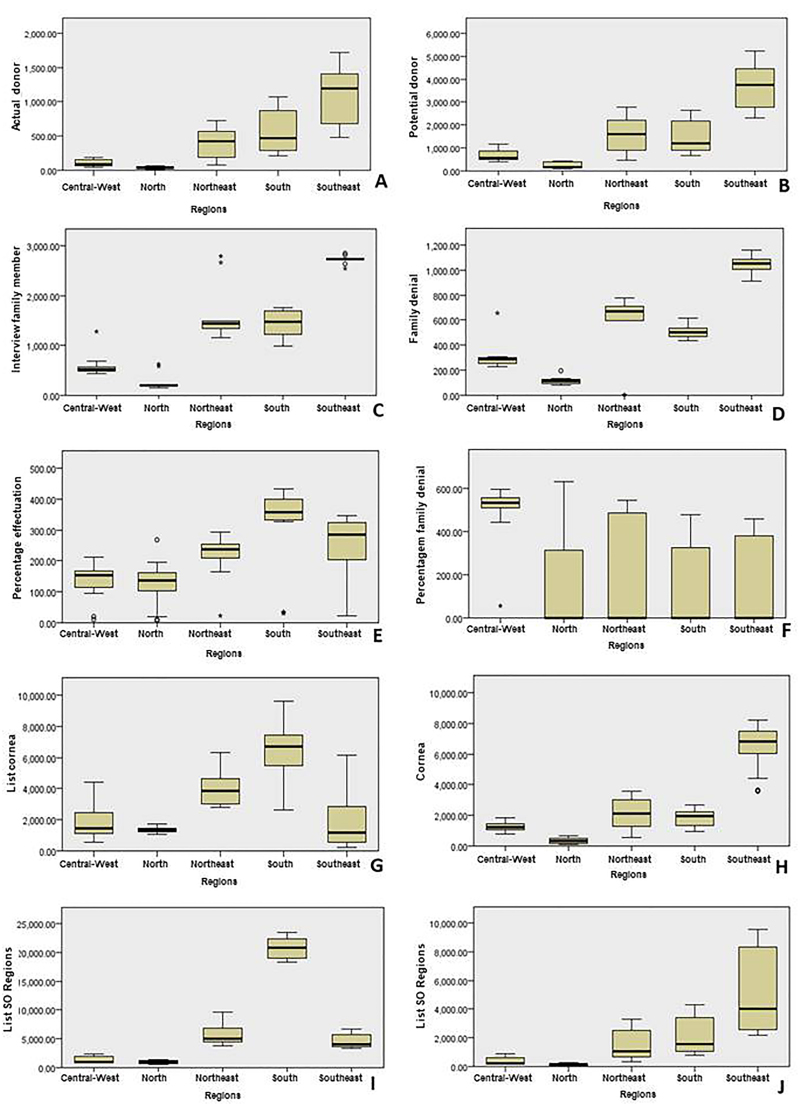
Demographic characterization of data related to solid organ and cornea transplantation by region of the country between 2008 and 2023 – Campo Grande, MS, Brazil, 2024.

**Legend:** SO = Solid organs.

**Note:** (A) Difference between the number of effective donors: North and Central-West < Northeast, South and Southeast; Northeast < Southeast (H = 85.807 (gl = 4); p < 0.001^1^); (B) Difference between the number of potential donors: Central-West and North < All regions; Northeast > Central-West and North; Northeast < Southeast; South > Central-West and North; South < Southeast; Southeast > All regions (F = 100.498 (gl = 4); p < 0.001^2^); (C) Difference between the number of family interviews carried out: North > South, Northeast and Southeast; Central-West > Southeast (H = 36.745 (gl = 4); p < 0.001^1^); (D) Difference between the number of family denials: North > Northeast, Southeast; Central-West > Southeast (H = 32.440 (gl = 4); p < 0.001^1^); (E) Difference between the percentage of completion: Central-West and North < Northeast, South and Southeast; North East > Central-West, North and South; South > Central-West, North and Southeast; Southeast – Central-West and North (F = 17.637 (gl = 4); p < 0.001^2^); (F) Difference between the percentage of family denial: North > Northeast, Southeast; Central-West > Southeast (H = 32.440 (gl = 4); p < 0.001^1^); (G) Difference in the cornea waiting list: North > Northeast, South; Southeast > Northeast, South; Central-West > Northeast, South (H = 40.286 (gl = 4); p < 0.001^1^); (H) Difference in the number of corneal transplants: North > All regions; Central-West > Southeast; Southeast > South, Northeast (H = 85.063 (gl = 4); p < 0.001^1^); (I) Difference between the number of people on the waiting list for solid organs: North > Southeast, Northeast, South; Central-West > Northeast, Southeast, South; Southeast > South (H = 60.815 (gl = 4); p < 0.001^1^); (J) Difference between the total number of solid organ transplants: North and Central-West > Northeast, South, Southeast; North East > Southeast (H = 82.616 (gl = 4); p < 0.001^1^); ^1^ Kruskal Wallis test with Dunn post-hoc test; One-way Anova test with Welch correction and Games-Howell post-hoc test.

According to data from the *SNT* between 2001 and 2021, 132,943 SO transplants and 250,799 cornea transplants were performed in Brazil, with emphasis on the southeast region, with 75,053 SO transplants and 134,140 cornea transplants. No data on cell donation in the period was found. The region with the lowest absolute number of transplants was the North region with 1,521 and 6,956 SO and cornea transplants, respectively (Figure 2).

Regarding costs, those related to SO transplants during the study period totaled R$6,618,744,901.88 and the total costs of procedures related to transplants totaled R$8,249,630,828.74. In 2023, until the month of April, R$ 146,365,455.94 were paid for transplants performed and a total of R$ 240,797,424.75 for procedures related to transplants, with an average of 14.6 days of hospitalization for SO transplantation.

In relation to the subgroups of procedures, the “collections of samples and exams for the purpose of donating organs, tissues and cells and of transplants” accounted for R$ 48,931,959.36 of the payment for transplant procedures, the “actions related to donation of organs and tissues for transplantation” for R$ 572,949,508.11 and “transplantation of organs, tissues and cells” for R$ 6,618,744,901.88 of the amount paid by the *SUS* (Table 1).

**Table 1:** Mean and standard deviation of subgroups of procedures related to transplantation per hospitalization, costs, days of stay and statistical difference in relation to the five regions of the country between the years 2008 and 2023 – Campo Grande, MS, Brazil, 2024.

Variable		n^(6)^	Mean (Standard deviation)	Statistics (gl)	p^(7)^
Collection and exams^(1)^	Hospitalization	64	152,111 (67,439)	46,746^(3)^	<0.001
	Costs^(4)^	64	764,561.86 (849,400.41)	4,046^(3)^	<0.001
	Permanence^(5)^	64	470.95 (589.44)	42,658^(3)^	<0.001
Related actions^(2)^	Hospitalization	85	3,394.13 (3,464.93)	64,532^(4)^	<0.001
	Costs^(4)^	80	7,161,868.85 (7,115,212.01)	68,699^(4)^	<0.001
	Permanence^(5)^	80	1,821.89 (1,810.44)	71,027^(4)^	<0.001
Transplants^(3)^	Hospitalization	85	2,273.56 (2,347.04)	62,580^(4)^	<0.001
	Costs^(4)^	80	82,734,311.28 (88,773,202.42)	70,760^(4)^	<0.001
	Permanence^(5)^	64	29,641.48 (26,003.30)	55,027^(3)^	<0.001

**Note**: ^1^collection and examinations for the purpose of donating organs, tissues and cells and of transplantation;

^2^actions related to the donation of organs and tissues for transplantation;

^3^transplantation of organs, tissues and cells;

^4^in reais;

^5^in days;

^6^months;

^7^Kruskal-Wallis test.

There were 9,735 hospitalizations for “sample collection and examinations for the purpose of donating organs, tissues and cells and transplantation” with a total of 30,141 days of hospitalization, 287,588 for “actions related to the donation of organs and tissues for transplantation” in 145,751 days and 192,545 for “organ, tissue and cell transplantation” which totaled 1,980,055 days of hospitalization. A statistically significant difference can be observed among the three variables and the regions of the country.

**Figure 3:**
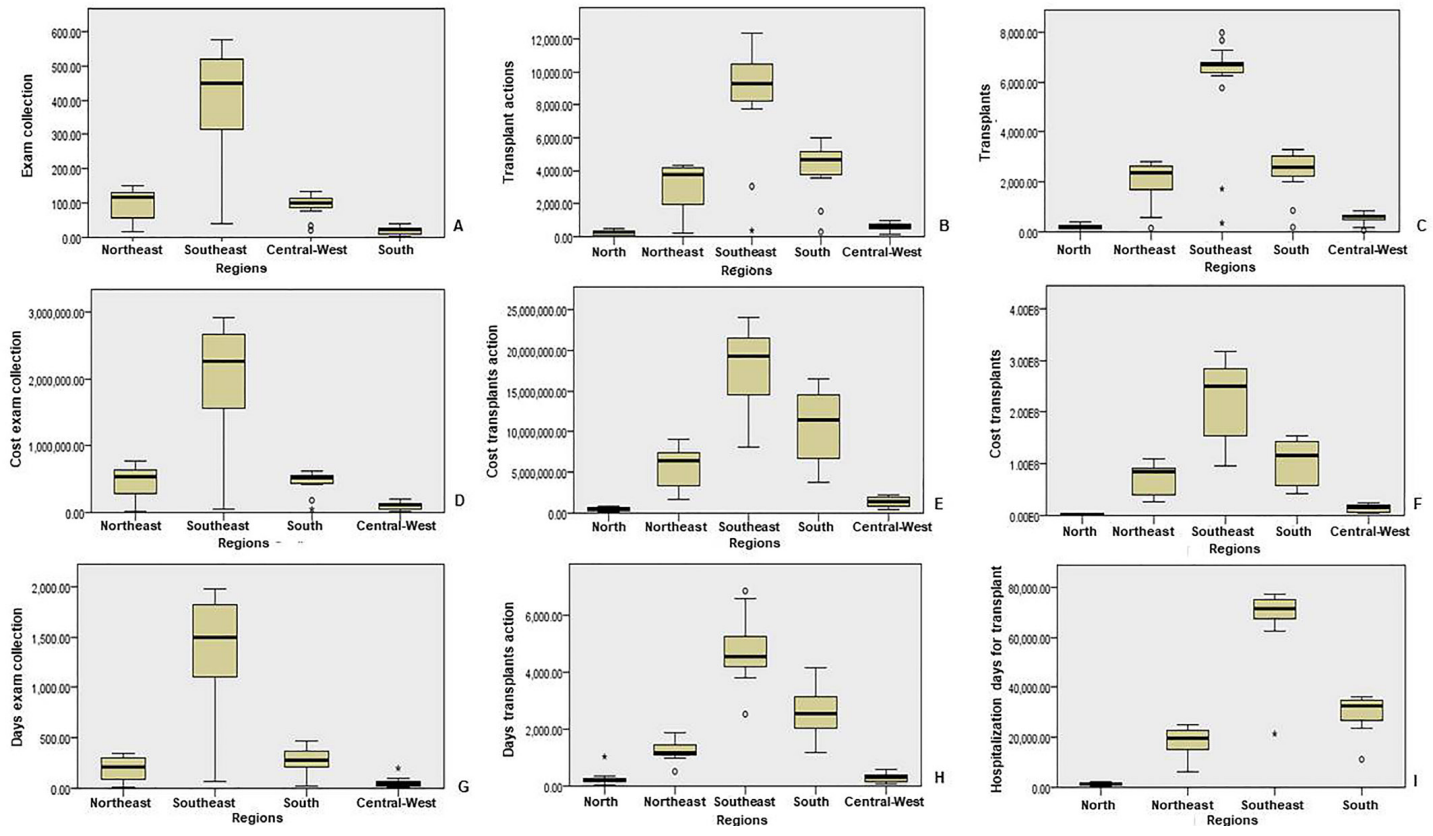
Comparison of subgroups of procedures related to transplantation by hospitalization, costs, and days of stay in the five regions of the country between the years 2008 and 2023 – Campo Grande, MS, Brazil, 2024.

**Note:** (A) Hospitalizations for collection and examinations for the purpose of donating organs, tissues and cells, and of transplantation: Central-West< South, Northeast and Southeast (p = 0.014; p = 0.002; p < 0.001); Southeast > South (p = 0.003); North East < Southeast (p = 0.018). (B) Hospitalizations for Actions related to the donation of organs and tissues for transplantation: North < Northeast, Southeast and South (p < 0.001); Central-West < South and Southeast (p = 0.005; p < 0.001); Northeast < Southeast (p = 0.024); (C) Hospitalizations for organ, tissue and cell transplantation: North < Northeast, Southeast and South (p < 0.001); Central-West < South and Southeast (p = 0.017; p < 0.001); Northeast < Southeast (p = 0.046); (D) Costs related to collection and exams for organ, tissue and cell donation and for transplantation: Central-West < Southeast, South and Northeast (p < 0.001; p = 0.020; p = 0.006); Southeast > South and Northeast (p = 0.007; p = 0.023); (E) Costs for Actions related to the donation of organs and tissues for transplantation: Southeast > Northeast (p = 0.010); North < Northeast, South, Southeast (p = 0.001; p < 0.001; p < 0.001); Southeast and South > Central-West (p < 0.001; p = 0.001); (F) Costs related to organ, tissue and cell transplantation: Southeast > Northeast (p = 0.016); North < Northeast, South and Southeast (p < 0.001); South and Southeast > Central-West (p = 0.003; p < 0.001); (G) Days of hospitalization for collection and examinations for the purpose of organ, tissue and cell donation and transplantation: Southeast –South and Northeast (p = 0.035; p = 0.001); Central-West – South, Southeast (p < 0.004; P < 0.001). (H) days of hospitalization for actions related to the donation of organs and tissues for transplantation: North < Northeast, South and Southeast (p = 0.013; p < 0.001; P < 0.001); Central-West < South and Southeast (p < 0.001); Northeast < Southeast (p = 0.002); (I) days of hospitalization for organ, tissue and cell transplantation: North < South and Southeast (p < 0.001); Northeast < Southeast (p < 0.001); Kruskal-Wallis test with Dunn Post-Hoc test.

Figure 3 shows the comparison between the subgroups of procedures related to transplants and Brazilian regions.

The correlation of data analyzed in this period was negative between the national waiting list and the total number of organ and tissue transplants performed, where the greater the number of transplants, the shorter the waiting list (r = –0.56; p = 0.036). The correlation between PD and AD was strongly positive (r = 0.97; p < 0.001) when the increase in the number of PD correlated with a greater number of AD. Likewise, the family interview was positively correlated with family denial (r = 0.72; p < 0.001), where the increase in the number of interviews was related to an increase in the number of family denials.

## DISCUSSION

Transplant procedures may be the only possibility of cure for patients with organ, tissue and cell failure. In the Brazilian context, the analysis of records from the National Hospital Information System carried out by this study demonstrates that there is a disparity in the number of transplants performed in each region. These differences may be related to economic, cultural, and logistical conditions that directly affect investment policies and the donation-transplantation process in each location^(1)^.

In 2022, the country had 1,944 hospitals with a capacity of more than 80 beds, that is, medium to large; however, only 469 Intra-Hospital Committees for Donation of Organs and Tissues for Transplants (CIHDOTT) and 61 Organ Procurement Organizations (OPO) were found, with the majority of these units located in the Southeast and South regions^(13)^.

Notably, the state of São Paulo hosts 66.6% of Brazilian transplant centers^(14)^, and this was reflected in the transplantation capacity of the Southeast region. In 2022, the state alone performed more than 10 thousand SO transplants^(2,14)^, which contrasts with the North region, which in comparison recorded the lowest absolute number of SO transplants in the country, with a total of 1,521 procedures in the same period.

The North and Central-West regions, in addition to having a low number of SO transplant centers, operate primarily for kidney procurement and transplantation^(1)^, and do not perform, for example, lung and pancreas transplants due to the lack of accredited services. These different realities contributed to the large difference in the number of transplants performed in the Southeast region found in the study.

Although the *SNT* is present throughout Brazil, logistical challenges, extensive geographic areas, and sparsely populated locations, as occurs in the North, restrict adequate infrastructure and hinder the process of procurement and provision of transplants to the population in these areas^(3)^. These obstacles limit access to adequate care and result in the migration of individuals to regions with greater availability of resources. Consequently, this generates inequalities in these essential services and highlights existing regional disparities.

Regarding financial aspects, this study observed that more than six billion reais in resources were spent on procedures and actions related to transplants between 2001 and 2021. To enhance the receipt of amounts by public-private institutions providing this type of assistance, the Ministry of Health adopted a reimbursement strategy through the Strategic Actions and Compensation Fund with the codification of procedures that are available at *SIH/SUS*. Thus, with each authorized hospitalization, the *SUS* can cover institutional debts^(15)^.

The evaluations carried out justify the financial expenses and regional efforts to achieve progress in these processes, as transplantation is the least expensive intervention from the perspective of the user’s life cycle for the health system^(16)^. In the country, the unit values of actions taken in transplantation are divided into hospital and professional services. In the kidney transplantation after donor brain death (BD), for example, the total value attributed to the procedure is R$ 27,622.67, with R$ 19,333.11 for the institution’s costs and R$ 8,289.56 for professionals involved in the process^(15)^. In the United States of America (USA), for the acquisition of the same type of organ, the total cost can reach up to US$35,542^(17)^.

When evaluating 482 medical records from a public hospital in São Paulo, a cost of US$6,064,986.51 was identified for the clinical maintenance of patients with chronic diseases. Of these, 67.6% were used in hospital care for individuals with chronic liver disease. Thus, the costs of more seriously ill patients may exceed the costs of liver transplantation^(18)^, considered theoretically more costly to the health system^(17)^. This occurs because the longer the waiting time for an organ, the greater the costs resulting from hospitalizations and procedures for the care of these individuals.

In five years, Brazil performed 9,823 kidney transplants, which generated a financial impact of more than R$588.3 million^(16)^. However, when comparing this organ transplantation with dialysis therapies, it is possible to consider very significant financial savings, ranging from R$ 5.9 to R$ 13.2 billion, which occurs for treatment by hemodialysis and peritoneal dialysis, respectively^(19)^. The cost reduction for services when carrying out transplants also occurred in another country. In a study conducted in the USA, performing the transplant generated savings of US$150,000.00^(20)^.

The economic theory of transplants is still in its early stages of growth and requires more details to imply in the formulation and implementation of public policies^(15)^. The values at all stages are considered quite high and the financial analysis of carrying out these procedures occurs, most of the time, from the perspective of direct costs. To understand the real financial impact, it is necessary to recognize, quantify, and value all resources used directly and indirectly with expenses in any organ acquisition^(17)^. Furthermore, there are important qualitative variables that can impact this financial measurement. Cost analyses combined with survival and quality of life rates are capable of generating important data for public health services and cooperating with the efficient use of resources that are limited^(19)^.

This research observed a moderate negative correlation between the waiting list and the total number of organs and tissues transplanted, but the trend of patients waiting for transplants was increasing. Although replacement therapies reach positive results in the country, the volume of procedures is still not capable of meeting the needs of the population, which is reflected in the growing waiting list for organs, cells and tissues^(21)^.

In 2022, the donation completion rate was 26.9%, 20% lower than the previous year. Furthermore, there was a low rate of BD notifications with a reduction of 18% this year. PD notification was 13,195 pmp/year; however, the AD record was 6,423 pmp/year with a donation of 3,528^(14)^. The conversion to AD is still insufficient for the country’s demand with the progressive annual increase in the waiting list.

In this context, the waiting list for a kidney takes the lead when compared to other organs. The mean waiting time adjusted for mortality is 5.5 years. Without this estimate, the time is approximately 11.1 years^(19)^, what means that many lives are lost each year because there are not enough organs for all patients^(22)^.

In the country, the supply of an organ is not inversely proportional to the performance of a transplant and many factors hinder the positive movement of the waiting list, which in 2022 closed with 52,989 patients^(14)^. In a hemodialysis treatment center in the Southeast region, 12,415 patients were found and of these, 77.2% did not undergo transplantation^(4)^.

There are several weaknesses found in the *SNT*. Among several factors, we can highlight the lack of care management strategies, especially with the diagnosis of BD, conduction of the donation process, ability to maintain the PD, and the insecurity in dealing with communication with the family and the mourning process^(2)^.

Countries such as Spain, Portugal and the USA have been successful in comparison to Brazil, mainly due to directing their resources to train teams to carry out all stages of the donation-transplantation process^(1)^, as well as to improve care structures and investments in quality programs that accompany opportunities for progress in the stages of this process^(1,2)^.

In this study, the family interview strongly correlated with family denial, which corroborates the increasing trend in the number of PD and decreasing number of AD. A PD’s diagnosis of BD is judicious and safe; however, the family assumes the relevant role in the ethical and legal prerogative of making this PD an AD^(22)^.

Family insecurity and low credibility in the health system, combined with cultural and religious factors, result in organ donations not being carried out in the country^(23,24)^. The absence of open and transparent dialogue in life has been a historical obstacle to family acceptance. Postponing this decision until the death of a loved one represents a challenge in family acceptance for donation, as the grieving process can influence this decision^(25)^.

The role of the family in the process of donating organs and tissues is decisive in improving the realities encountered and the lack of family authorization is the main reason why an organ is not donated in Brazil and in several countries abroad, with rates ranging from 5.7 to 41.4% in European countries, and 27.5 to 48.9% in Latin American countries. In Brazil, a study indicates a variation percentage of 37.3% to 70% depending on the region^(26)^.

Among the refusal prerogatives, those with the highest rates found in the studies are related to keeping the body intact (36.0%) and insecurity about the donation process (32.6%)^(26)^. In order for these obstacles to be resolved and the number of acceptances for organ and tissue donations to increase, a priority investment is required, in the context of the family approach when formulating new public policies^(9)^, in the training of the professionals involved, and finally, in transparency with the outcomes of the entire process made publicly available^(23)^.

The nursing team, as well as the multidisciplinary team, plays a fundamental role in the family approach and their conduct can be a decisive point in the acceptance of the donation. Through their work, nursing professionals can be a link between the health service and family members by offering support in a moment of emotional vulnerability with sensitive communication, active and qualified listening, grief support, provision of guidance and related clarifications to the procedure^(27)^.

Another important point concerns the need for society to be aware of organ donation. As a strategy, social marketing is essential in this context, as it encourages reflection on its importance, promotes family dialogue, and can encourage donation consent^(26)^. Carrying out a transplant is of incalculable value for the recipient, their family members, and also society in general, as in addition to restoring the individual’s autonomy, it reduces the costs of health services and has the potential to return this individual to the labor market.

Therefore, it is noticeable that the costs associated with transplants exceed the available values recorded on official platforms. It is essential to consider the social and economic factors of individuals of working age, who, due to their health condition, are outside the labor market, impacting the national social security sector. Furthermore, the countless hospitalizations, clinical and medication treatments burden the health sector and directly interfere with the resources allocated to this area.

From this perspective, it can be understood that the information presented by this study provides relevant evidence about the donation and transplantation process, which is extensive, complex and needs to be effectively guided, with the possibility of reviewing workflows and management models, aiming at better management and allocation of resources. Assessing care and financial results has been a challenge for health services^(2)^, but they constitute a vital point in the management of scarce investments in search of the best results.

### Limitations

Regarding the methodological limitations of the study, the use of secondary data in the public domain should be highlighted, which may introduce an information bias due to the lack of control over the quality of evaluation and measurement of these data. Furthermore, important confounding variables such as coordination and logistics capacity, regional hospital infrastructure, population awareness about organ donation, and specific local policies were not included in the information sources, which could have an impact on the results of the analysis. These issues were discussed in the study, but their non-inclusion may have influenced the results in general.

In addition to methodological limitations, it is also important to recognize other limitations. For instance, the lack of budgetary data regarding the indirect costs of transplants may have limited a complete understanding of the financial impact of these procedures. Furthermore, the scarcity of research on the topic limited the evidence base available to support the study’s discussions.

### Contributions to the Health Area

The study shows that the costs related to transplant procedures go beyond officially reported values and provides a valuable contribution to public health managers by signaling the need to consider the direct financial aspects and individual social and economic factors of patients on the waiting list. In addition, as this is a mixed ecological study that uses the population aggregate of Brazilian regions, it is possible to identify more precisely which regions face greater limitations in transplant procedures. This, in its turn, highlights the importance of more effective investments and public policies to serve a greater number of individuals in these regions.

## CONCLUSION

The numbers related to transplant procedures carried out in Brazil contrast with the waiting list for a solid organ or tissue, which occurs in greater numbers in the South and Northeast regions of the country. Procurement and transplantation logistics and the gross domestic product of each region can contribute to increasing these numbers. Moreover, the number of family denials identified as the main reason for non-donation of organs and tissues is associated with an increase in the waiting list for an organ or tissue.

This difference between the number of transplants within the Brazilian regions reflects the need for organization and new accreditation of transplant centers, as well as greater dissemination of information about BD and transplantation to the population, aiming at raising awareness on the importance of organs, cells, and tissues donation.
