# Supplementary material for: Transplant management in Brazil: a temporal analysis of financial investments and procedures
Source: Rev Esc Enferm USP. 2024 Aug 23;58:e20240039. doi: 10.1590/1980-220X-REEUSP-2024-0039en (PMC11349334; doi:10.1590/1980-220X-REEUSP-2024-0039en)
Supplement: Translation

# Translation: Gestão de transplantes no Brasil: análise temporal dos investimentos financeiros e procedimentos

**DOI:** 10.1590/1980-220X-REEUSP-2024-0039pt

**Authors:** Mercy da Costa Souza, Marcos Antonio Ferreira, Carolina Mariano Pompeo, Felipe Machado Mota, Elenir Rose Jardim Cury

**Affiliations:** 1 Universidade Federal de Mato Grosso do Sul, Programa de Pós-Graduação em Saúde e Desenvolvimento na Região Centro-Oeste. Campo Grande, MS, Brasil.; 2 Universidade Federal de Mato Grosso do Sul, Programa de Pós-Graduação em Enfermagem do Instituto Integrado de Saúde. Campo Grande, MS, Brasil.

**Keywords:** Transplante de Órgãos, Obtenção de Tecidos e Órgãos, Custos e Análise de Custo, Acessibilidade aos Serviços de Saúde, Epidemiologia

## Abstract

**Objetivo:**

Analisar as ações gerenciais públicas dos investimentos financeiros e procedimentos de transplantes de órgãos, células e tecidos no Brasil.

**Método:**

Estudo ecológico misto (tempo e local), realizado com base em dados do Sistema de Informações Hospitalares do Sistema Único de Saúde do Departamento de Informática do SUS e do Sistema Nacional de Transplante, de 2001 a 2023. Foram realizadas análises de tendência temporal, estatística descritiva e inferencial.

**Resultados:**

Os transplantes de órgãos, células e tecidos encontram-se concentrados na região Sudeste do país, com aumento dos custos no local. As regiões Nordeste e Sul do Brasil apresentam a maior fila de espera, com uma tendência crescente (R^2^ = 0,96), associada a tendência decrescente do número de transplantes (R^2^ = 0,97).

**Conclusão:**

A diferença do total de transplantes e procedimentos realizados entre as regiões do Brasil representa a necessidade de organização e investimentos com estratégias voltadas para a capacitação de profissionais e conscientização da população.

## INTRODUÇÃO

A demanda por transplantes cresce consideravelmente em todo mundo devido ao aumento das doenças crônicas e degenerativas não transmissíveis, como diabetes, hipertensão e câncer, entre outras causas. Essa terapia substitutiva visa proporcionar melhorias clínicas para indivíduos cujas condições de saúde não respondem adequadamente a tratamentos convencionais ou menos invasivos^(1–3)^.

A Organização Mundial da Saúde relata que cerca de 130.000 transplantes de órgãos sólidos (OS) são realizados por ano, o que representa apenas 10% da necessidade mundial. Entretanto, a estimativa é de que no Brasil essa taxa possa chegar até 30%, devido ao seu grande programa público, o Sistema Nacional de Transplantes (SNT)^(4)^.

Para que o processo de doação e transplantação seja efetivado no Brasil, o SNT, sob as diretrizes do Ministério da Saúde (MS), opera conforme a Lei nº 9.434/1997, que proporciona um suporte fundamental e determina que o SNT regulamente, coordene e supervisione toda a rede de atividades do sistema público de transplantes no território nacional^(5,6)^.

O financiamento e a gestão dos transplantes variam ao redor do mundo^(7,8)^. Enquanto países da América Central lidam com sistemas de saúde fragmentados e heterogêneos, com financiamento parcial do governo^(9,10)^, o Sistema Único de Saúde (SUS) do Brasil é reconhecido internacionalmente por oferecer assistência integral e gratuita pré e pós-transplante, com mais de 90% dos procedimentos custeados pelo poder público^(3,5,6)^.

Embora o SUS financie os procedimentos relacionados a transplantes e o programa público brasileiro seja reconhecido internacionalmente^(11)^, a quantidade de terapias substitutivas realizadas ainda não atende à demanda da população. Em dezembro de 2023, apenas 15% dos pacientes em lista de espera para transplantes foram atendidos, o que resultou em 59.958 indivíduos que, ao final do ano, ainda aguardavam a transplantação^(12)^.

Esse elevado número de pessoas em fila de espera varia entre as regiões brasileiras e representa as diferentes capacidades de investimento e gestão de cada uma. Devido às características continentais do Brasil, suas cinco regiões – Norte, Nordeste, Sudeste, Centro-Oeste e Sul – apresentam particularidades geográficas, demográficas e socioeconômicas distintas^(11)^. Essas diferenças resultam em níveis variados de investimento em saúde e influenciam diretamente a logística, infraestrutura, capacidade de aquisição de órgãos e transplantação de cada local.

O transplante é um tratamento altamente eficaz para falência avançada de órgãos e muitas vezes consiste na única opção terapêutica indicada para prolongar a vida do indivíduo^(1)^. À medida que o número de doenças crônicas não transmissíveis, com efeitos potencialmente prejudiciais aos órgãos, continua a crescer, a demanda por esse serviço inevitavelmente aumentará^(3)^.

Com isso, caso não haja uma gestão com investimentos eficientes, de acordo com as particularidades de cada região, a lista única, que já conta com uma grande quantidade de pacientes, passará a abranger cada vez mais pessoas que necessitam deste procedimento^(1)^. Portanto, é essencial determinar o perfil de investimento e os procedimentos realizados em cada região para entender como as políticas de gestão do poder público podem impactar a eficiência dos serviços de transplantação no país.

Adicionalmente, a literatura brasileira apresenta uma lacuna significativa de evidência em relação aos desafios sistêmicos, infraestruturais e geográficos enfrentados pelo SUS, que podem comprometer a capacidade de acesso dos indivíduos aos serviços de transplante e à obtenção de tratamentos de alta qualidade^(3)^. Diante dessa realidade, o presente estudo tem como objetivo analisar as ações gerenciais públicas dos investimentos financeiros e procedimentos de transplantes de órgãos, células e tecidos no Brasil.

## MÉTODO

### Desenho do Estudo

Estudo epidemiológico, de delineamento ecológico misto (tempo e lugar), realizado por meio de dados secundários do Sistema de Informações Hospitalares do SUS (SIH/SUS) do Departamento de Informática do SUS (DATASUS) e dos relatórios estatísticos do SNT.

### Local, População e Critério de Seleção

O estudo ecológico misto foi realizado com dados de agregados populacionais das cinco regiões brasileiras, Centro-Oeste, Norte, Nordeste, Sudeste e Sul, os quais foram obtidos de duas fontes de informações distintas: o SIH/SUS e os relatórios estatísticos do SNT. A definição da população do estudo foi realizada mediante as características individuais de cada base.

Para os dados do SIH/SUS, foram incluídos todos os registros de internações hospitalares custeadas pelo SUS no período de 2008 a 2023. Já em relação aos dados dos relatórios estatísticos do SNT, a população utilizada foi derivada dos transplantes e da doação de órgãos, tecidos e células realizados no período de 2001 a 2021, bem como da lista de espera referente ao período de 2008 a 2021. O recorte temporal para ambas as bases de dados foi definido conforme a disponibilidade dos dados durante o período de coleta.

A seleção dos dados obtidos por meio do SIH/SUS foi definida pela aplicação do filtro de região/unidade da federação, ano de processamento (2008 a 2023), procedimentos (número de internações, custos e dias de internação referentes aos transplantes), e subgrupo de procedimentos realizados para o transplante. Já para os relatórios do SNT foram incluídos todos os dados disponibilizados no documento.

O uso de duas fontes de informação distintas para definir a população deu-se em razão de abranger e detalhar o objeto de estudo, uma vez que enquanto o SIH/SUS fornece informações mais específicas como quantidades de internação, dias de internação, valor e procedimentos relacionados a transplantes, os relatórios do SNT fornecem números referentes a quantidade de doadores, pacientes em fila de espera, porcentagem de negativa familiar e, por fim, a quantidade de transplantes efetivados.

### Coleta de Dados

A coleta de dados foi realizada no período de 01 a 31 de julho de 2023. Todas as informações relacionadas ao transplante de órgãos, tecidos e células no período de janeiro de 2008 a março de 2023 foram extraídas via internet do SIH/SUS com utilização do TabNet Win32 3.0. Os dados coletados foram referentes ao número de internações, custos e dias de internação para procedimentos relacionados ao transplante.

As variáveis coletadas foram: total de internações para transplante de órgãos, tecidos e células, valor total do transplante, média de dias de internação para os procedimentos de transplantes. Além destes, foram incluídos dados referentes aos subgrupos de procedimentos realizados para o transplante: coleta e exames para fins de doação de órgãos, tecidos e células e de transplantes; ações relacionadas à doação de órgãos, células e tecidos para transplante; e transplante de órgãos, tecidos e células.

Em um segundo momento foram obtidos os dados dos relatórios do SNT relacionados ao transplante e doação de órgãos, tecidos e células do período de 2001 a 2021 e da lista de espera de 2008 a 2021. As variáveis coletadas do SNT tratam do número total de pacientes em fila de espera de órgãos e tecidos; quantidade de transplantes de órgãos sólidos (OS) e córneas, em número absoluto e por milhão de população (pmp); total de potencial doador (PD) e doador efetivo (DE); lista de espera por OS e córneas; número absoluto e percentual de negativa familiar e de efetivação da doação de órgãos. Após o período de coleta, as informações foram exportadas para uma planilha do *software Microsoft Excel* ^®^ para realizar a tabulação.

### Análise e Tratamento de Dados

Os dados foram analisados de forma individual de acordo com cada fonte adotada, ou seja, não houve cruzamento dos dados entre SIH/SUS e relatórios estatísticos do SNT. As análises descritivas e inferências foram realizadas de acordo com as características dos dados de cada uma. Dados faltantes não foram considerados para a análise inferencial.

Foi realizada uma análise de tendência temporal para verificar o comportamento histórico das variáveis investigadas por meio da técnica de média móvel simples (MMS) calculada em ciclos de três anos usando a fórmula (MMS = (P1 + P2 + P3)/3). Posteriormente, gráficos temporais foram construídos para representar o número total de pacientes em fila de espera especificamente por OS, assim como para lista de espera por uma córnea. Além disso, o número total de transplantes e transplantes por pmp, o número de transplantes de OS e de córnea pmp e do total de PD e DE também foram considerados para verificar o possível formato da curva de tendência a ser estudada.

Após esse processo, foram aplicados os modelos de regressão polinomial, de modo que o modelo que mais se adequou à curva foi o de terceiro grau, também chamado de cúbico e representado pela fórmula (Y = β_0 + β_1 X + β_1 X^2 + β_1 X^3). Os modelos foram escolhidos de acordo com o maior coeficiente de determinação (R^2^). Os desfechos primários utilizados na análise de tendência foram: a tendência de aumento ou declínio ao longo do tempo do número – absoluto e pmp – de transplantes e de pacientes na fila de espera e número absoluto de potenciais doadores.

Para análise dos procedimentos relacionados ao transplante foi realizada análise inferencial. O teste de *Shapiro-Wilk* foi aplicado para verificação da normalidade de distribuição dos dados. Após foi aplicado o teste Anova de uma via para as variáveis com distribuição normal e o teste de *Kruskal-Wallis* caso o pressuposto da normalidade não tenha sido acatado. Os testes de *Post-Hoc* de *Games-Howell* e de *Dunn* foram utilizados para avaliar a diferença estatística encontrada entre as regiões.

Nas análises inferenciais de grupos foram avaliados os seguintes desfechos primários: a diferença – em número absoluto – de doadores efetivos; entrevistas e negativas familiares; percentual de efetivação; lista de espera de córnea e de OS; transplantes de córnea e OS entre as regiões do Brasil. Todos os dados foram retirados dos relatórios do SNT.

Em relação à análise de grupos dos dados retirados do SIH/SUS os desfechos primários estudados foram: o número de internações; custo em reais e dias de permanência para coleta e exames para fins de doação de órgãos, tecidos e células e de transplantes; ações relacionadas à doação de órgãos e tecidos para transplante; e transplante de órgãos, tecidos e células entre as regiões do Brasil.

Para verificar a correlação entre as variáveis foram realizados os testes de *Pearson* para variáveis paramétricas e *Spearman* para as não paramétricas. Para todos os testes foi utilizado o nível de significância de 0,05.

### Aspectos Éticos

Por se tratar de dados de domínio públicos e não nominais, não houve necessidade de submissão e aprovação prévia por um Comitê de Ética em Pesquisa com Seres Humanos.

## RESULTADOS

Em relação à lista de espera para transplante obtida por meio dos dados do SNT de 2008 a 2021, 463.637 pacientes aguardaram por um órgão sólido e 213.823 pelo transplante de córnea em algum momento deste período, com média de 33.116 (DP = 4.328,88) e 15.273,07 (DP = 5.244,99), respectivamente. Os anos com o maior número de pessoas na espera por um órgão foram 2008 e 2009, com 64.275 e 63.866 cada.

A média de PD ao longo do período foi de 385,27 (DP = 190,53) e do DE encontrado foi de 100,11 (DP = 78,45) pmp. Foi realizada uma média de 1.341,67 (DP = 928,86) entrevistas com a família do PD, com média de 504,88 (DP = 345,27) recusas à doação de órgãos e tecidos. A Figura 1 apresenta a média móvel e tendência temporal dos dados relacionados ao transplante entre os anos de 2001 e 2021.

**Figura 1:** Curva de tendência temporal e média móvel da fila de espera por transplantes de 2008 a 2021, número de transplantes e número de transplantes por milhão de população (órgãos sólidos e córneas), número de potenciais doadores e doadores efetivos de 2001 a 2021 – Campo Grande, MS, Brasil, 2024.

**Legenda:** MM = Média móvel; OS = Orgãos sólidos; pmp = Por milhão de população.

**Nota:** (A) Média móvel e curva de tendência da fila de espera de órgãos sólidos e córnea (R^2^ = 0,96); da fila de espera de órgãos sólidos (R^2^ = 0,94); da fila de espera de córnea (R^2^ = 0,91); (B) Média móvel e curva de tendência do número de transplantes de órgãos sólidos e córnea (R^2^ = 0,97); do número de transplantes de órgãos sólidos (R^2^ = 0,94); do número de transplantes de córnea (R^2^ = 0,98); (C) Média móvel e curva de tendência da taxa de transplantes de órgãos sólidos por milhão de população (R^2^ = 0,93); da taxa de transplantes de córnea por milhão de população (R^2^ = 0,98); (D) Média móvel e curva de tendência do número de potenciais doadores (R^2^ = 0,99) e doadores efetivos (R^2^ = 0,99).

De acordo com os dados do SNT no período compreendido entre 2001 a 2021, foram realizados 132.943 transplantes de OS e 250.799 transplantes de córnea no Brasil, com destaque para a região sudeste, com 75.053 transplantes de OS e 134.140 transplantes de córneas. Não foram encontrados dados sobre a doação de células no período. A região com menor número absoluto de transplantes foi a região Norte com 1.521 e 6.956 transplantes de OS e córnea, respectivamente (Figura 2).

**Figura 2:** Caracterização demográfica dos dados relacionados ao transplante de órgãos sólidos e córneas por região do país entre os anos de 2008 a 2023 – Campo Grande, MS, Brasil, 2024.

**Legenda:** OS = Órgãos sólidos.

**Nota:** (A) Diferença entre o número de doadores efetivos: Norte e Centro-Oeste < Nordeste, Sul e Sudeste; Nordeste < Sudeste (H = 85,807 (gl = 4); p < 0,001^1^); (B) Diferença entre o número de potenciais doadores: Centro-Oeste e Norte < Todas as regiões; Nordeste > Centro-Oeste e Norte; Nordeste < Sudeste; Sul > Centro-Oeste e Norte; Sul < Sudeste; Sudeste > Todas as regiões (F = 100,498 (gl = 4); p < 0,001^2^); (C) Diferença entre o número de entrevistas familiares realizadas: Norte > Sul, Nordeste e Sudeste; Centro-Oeste > Sudeste (H = 36,745 (gl = 4); p < 0,001^1^); (D) Diferença entre o número de negativas familiares: Norte > Nordeste, Sudeste; Centro-Oeste > Sudeste (H = 32,440 (gl = 4); p < 0,001^1^); (E) Diferença entre o percentual de efetivação: Centro-Oeste e Norte < Nordeste, Sul e Sudeste; Nordeste > Centro-Oeste, Norte e Sul; Sul > Centro-Oeste, Norte e Sudeste; Sudeste – Centro-Oeste e Norte (F = 17,637 (gl = 4); p < 0,001^2^); (F) Diferença entre o percentual de negativa familiar: Norte > Nordeste, Sudeste; Centro-Oeste > Sudeste (H = 32,440 (gl = 4); p < 0,001^1^); (G) Diferença entre a lista de espera de córnea: Norte > Nordeste, Sul; Sudeste > Nordeste, Sul; Centro-Oeste > Nordeste, Sul (H = 40,286 (gl = 4); p < 0,001^1^); (H) Diferença no número de transplantes de córnea: Norte > Todas as regiões; Centro-Oeste > Sudeste; Sudeste > Sul, Nordeste (H = 85,063 (gl = 4); p < 0,001^1^); (I) Diferença entre o número de pessoas na lista de espera por órgãos sólidos: Norte > Sudeste, Nordeste, Sul; Centro-Oeste > Nordeste, Sudeste, Sul; Sudeste > Sul (H = 60,815 (gl = 4); p < 0,001^1^); (J) Diferença entre o número total de transplantes de órgãos sólidos: Norte e Centro-Oeste > Nordeste, Sul, Sudeste; Nordeste > Sudeste (H = 82,616 (gl = 4); p < 0,001^1^); ^1^ Teste de Kruskal Wallis com post Hoc de Dunn; Teste Anova de uma via com correção de Welch e post Hoc de Games-Howell.

Quanto aos custos, os relacionados aos transplantes de OS no período de estudo totalizaram R$ 6.618.744.901,88 e os custos totais dos procedimentos relacionados aos transplantes somaram R$ 8.249.630.828,74. Em 2023, até o mês de abril foram pagos R$ 146.365.455,94 para os transplantes realizados e um total de R$ 240.797.424,75 para os procedimentos relacionados aos transplantes, com a média de 14,6 dias de internação para o transplante de OS.

Em relação aos subgrupos de procedimentos, as “coletas de amostras e exames para fins de doação de órgãos, tecidos e células e de transplantes” foram responsáveis por R$ 48.931.959,36 do pagamento pelos procedimentos de transplante, as “ações relacionadas à doação de órgãos e tecidos para transplante” por R$ 572.949.508,11 e o “transplante de órgãos, tecidos e células” por R$ 6.618.744.901,88 da quantia paga pelo SUS (Tabela 1).

**Tabela 1:** Média e desvio padrão dos subgrupos de procedimentos relacionados ao transplante por internação, custos, dias de permanência e a diferença estatística em relação às cinco regiões do país entre os anos de 2008 a 2023 – Campo Grande, MS, Brasil, 2024.

Variável		n^(6)^	Média (Desvio-padrão)	Estatística (gl)	p^(7)^
Coleta e exames^(1)^	Internação	64	152,111(67,439)	46,746^(3)^	<0,001
	Custos^(4)^	64	764.561,86 (849.400,41)	4,046^(3)^	<0,001
	Permanência^(5)^	64	470,95 (589,44)	42,658^(3)^	<0,001
Ações relacionadas^(2)^	Internação	85	3.394,13 (3.464,93)	64,532^(4)^	<0,001
	Custos^(4)^	80	7.161.868,85 (7.115.212,01)	68,699^(4)^	<0,001
	Permanência^(5)^	80	1.821,89 (1.810,44)	71,027^(4)^	<0,001
Transplantes^(3)^	Internação	85	2.273,56 (2.347,04)	62,580^(4)^	<0,001
	Custos^(4)^	80	82.734.311,28 (88.773.202,42)	70,760^(4)^	<0,001
	Permanência^(5)^	64	29.641,48 (26.003,30)	55,027^(3)^	<0,001

**Nota**: ^1^coleta e exames para fins de doação de órgãos, tecidos e células e de transplante;

^2^ações relacionadas à doação de órgãos e tecidos para transplante;

^3^transplante de órgãos, tecidos e células;

^4^em reais;

^5^em dias;

^6^meses;

^7^teste de Kruskal-Wallis.

Foram 9.735 internações para “coletas de amostras e exames para fins de doação de órgãos, tecidos e células e de transplante” com um total de 30.141 dias de internação, 287.588 para as “ações relacionadas à doação de órgãos e tecidos para transplante” em 145.751 dias e 192.545 para o “transplante de órgãos, tecidos e células” que totalizou 1.980.055 dias de internação. Pode ser observada diferença estatisticamente significativa entre as três variáveis e as regiões do país.

A Figura 3 apresenta a comparação entre os subgrupos de procedimentos relacionados aos transplantes e as regiões brasileiras.

**Figura 3:** Comparação dos subgrupos de procedimentos relacionados ao transplante por internação, custos e dias de permanência nas cinco regiões do país entre os anos de 2008 a 2023 – Campo Grande, MS, Brasil, 2024.

**Nota:** (A) Internações para coleta e exames para fins de doação de órgãos, tecidos e células e de transplante: Centro-Oeste< Sul, Nordeste e Sudeste (p = 0,014; p = 0,002; p < 0,001); Sudeste>Sul (p = 0,003); Nordeste < sudeste (p = 0,018). (B) Internações para Ações relacionadas à doação de órgãos e tecidos para transplante: Norte < Nordeste, Sudeste e Sul (p < 0,001); Centro-Oeste < Sul e Sudeste (p = 0,005; p < 0,001); Nordeste < Sudeste (p = 0,024); (C) Internações para Transplante de órgãos, tecidos e células: Norte < Nordeste, Sul e Sudeste (p < 0,001); Centro-Oeste < Sul e Sudeste (p = 0,017; p < 0,001); Nordeste < Sudeste (p = 0,046); (D) Custos relacionados à coleta e exames para fins de doação de órgãos, tecidos e células e de transplante: Centro-Oeste < Sudeste, Sul e Nordeste (p < 0,001; p = 0,020; p = 0,006); Sudeste > Sul e Nordeste (p = 0,007; p = 0,023); (E) Custos para Ações relacionadas à doação de órgãos e tecidos para transplante: Sudeste > Nordeste (p = 0,010); Norte < Nordeste, Sul, Sudeste (p = 0,001; p < 0,001; p < 0,001); Sudeste e Sul > Centro-Oeste (p < 0,001; p = 0,001); (F) Custos relacionados ao Transplante de órgãos, tecidos e células: Sudeste > Nordeste (p = 0,016); Norte < Nordeste, Sul e Sudeste (p < 0,001); Sul e Sudeste > Centro-Oeste (p = 0,003; p < 0,001); (G) Dias de internação para coleta e exames para fins de doação de órgãos, tecidos e células e de transplante: Sudeste –Sul e Nordeste (p = 0,035; p = 0,001); Centro-Oeste – Sul, Sudeste (p < 0,004; p < 0,001). (H) dias de internação para as ações relacionadas à doação de órgãos e tecidos para transplante: Norte < Nordeste, Sul e Sudeste (p = 0,013; p < 0,001; p < 0,001); Centro-Oeste < Sul e Sudeste (p < 0,001); Nordeste < sudeste (p = 0,002); (I) dias de internação para transplante de órgãos, tecidos e células: Norte < Sul e Sudeste (p < 0,001); Nordeste < sudeste (p < 0,001); teste de Kruskal-Wallis com Post-Hoc de Dunn.

A correlação dos dados analisados neste período mostrou-se negativa entre a lista de espera nacional e o total de transplantes de órgãos e tecidos realizados, onde quanto maior o número de transplantes, menor a lista de espera (r = –0,56; p = 0,036). Já a correlação entre o PD e o DE foi fortemente positiva (r = 0,97; p < 0,001) quando o aumento do número de PD se correlacionou com um maior número de DE. Da mesma forma, a entrevista familiar correlacionou positivamente com a negativa familiar (r = 0,72; p < 0,001), onde o aumento no número de entrevistas foi relacionado com um aumento no número de negativa familiar.

## DISCUSSÃO

Os procedimentos de transplantes podem consistir na única possibilidade de cura para pacientes com falência de órgãos, tecidos e células. No contexto brasileiro, a análise dos registros do Sistema Nacional de Informações Hospitalares realizada por este estudo demonstra que ocorre uma disparidade no número de transplantes realizados em cada região. Essas diferenças podem estar relacionadas com condições econômicas, culturais e logísticas que implicam diretamente nas políticas de investimento e no processo de doação-transplantação de cada local^(1)^.

Em 2022, o país contava com 1.944 hospitais com capacidade superior a 80 leitos, ou seja, médio a grande porte; porém, foram identificadas apenas 469 Comissões Intra-Hospitalares para Doação de Órgãos e Tecidos para Transplantes (CIHDOTT) e 61 Organizações de Procura de Órgãos (OPO), com a maioria dessas unidades localizadas nas regiões Sudeste e Sul^(13)^.

Notavelmente, o estado de São Paulo abriga 66,6% dos centros transplantadores brasileiros^(14)^, e isso refletiu na capacidade de transplantação do Sudeste. Em 2022, somente o estado realizou mais de 10 mil transplantes de OS^(2,14)^, o que contrasta com a região Norte, que em comparação registrou o menor número absoluto de transplantes de OS do país, com um total de 1.521 procedimentos no mesmo período.

As regiões Norte e Centro-Oeste, além de possuírem um baixo número de centros de transplantação de OS, atuam prioritariamente para a captação e transplante de rins^(1)^, e não realizam, por exemplo, o transplante de pulmão e pâncreas devido à ausência de serviços credenciados. Essas diferentes realidades contribuíram para que houvesse uma grande diferença no número de transplantes realizados pelo Sudeste encontrado no estudo.

Embora o SNT esteja presente em todo território brasileiro, desafios logísticos, extensas áreas geográficas e localidades pouco povoadas, como ocorre no Norte, restringem a infraestrutura adequada e dificultam o processo de captação e oferta de transplante para a população nessas áreas^(3)^. Esses obstáculos limitam o acesso ao atendimento adequado e resultam na migração de indivíduos para regiões com maior disponibilidade de recursos. Consequentemente isso gera desigualdades nesses serviços essenciais e evidencia as disparidades regionais existentes.

No que se refere aos aspectos financeiros, este estudo identificou que mais de seis bilhões de reais em recursos foram gastos em procedimentos e ações relacionadas aos transplantes entre 2001 a 2021. Para potencializar o recebimento dos valores pelas instituições público-privadas prestadoras deste tipo de assistência, o Ministério da Saúde adotou uma estratégia de reembolso pelo Fundo de Ações Estratégicas e Compensação com a codificação dos procedimentos que estão disponíveis no SIH/SUS. Assim, mediante cada internação autorizada, o SUS pode custear os débitos institucionais^(15)^.

As avaliações realizadas justificam as despesas financeiras e os esforços regionais para que se tenha uma evolução nesses processos, pois o transplante é a intervenção menos onerosa na perspectiva do ciclo vital do usuário para o sistema de saúde^(16)^. No país, os valores unitários das ações empregadas no transplante são divididos em serviços hospitalares e profissionais. No transplante renal do doador pós morte encefálica (ME), por exemplo, o valor total atribuído ao procedimento é de R$ 27.622,67, com R$ 19.333,11 para o custeio da instituição e R$ 8.289,56 para profissionais envolvidos no processo^(15)^. Nos Estados Unidos da América (EUA), para a aquisição do mesmo tipo de órgão, o custo integral pode chegar até US$ 35.542^(17)^.

Ao avaliar 482 prontuários de um hospital público de São Paulo foi identificado um custo de US$ 6.064.986,51 para a manutenção clínica de pacientes com doenças crônicas. Destes, 67,6% foram empregados na atenção hospitalar ao indivíduo com hepatopatia crônica. Assim, os custos com pacientes mais graves podem exceder os custos do transplante hepático^(18)^, considerado teoricamente mais oneroso ao sistema de saúde^(17)^. Isso ocorre pois quanto maior o tempo de espera por um órgão maiores serão os custos decorrentes de internações e procedimentos para a assistência desses indivíduos.

Em cinco anos, o Brasil realizou 9.823 transplantes renais, o que gerou um impacto financeiro superior a R$ 588,3 milhões^(16)^. No entanto, ao comparar o transplante deste órgão com as terapias dialíticas é possível considerar uma economia financeira bastante expressiva, com a variação de R$ 5,9 a R$ 13,2 bilhões, que se dá para o tratamento por hemodiálise e diálise peritoneal, respectivamente^(19)^. A redução de custos para os serviços ao efetivar os transplantes também ocorreu em outro país. Em um estudo conduzido nos EUA, realizar o transplante gerou uma economia de US$150.000,00^(20)^.

A teoria econômica dos transplantes ainda está em seus estágios iniciais de crescimento e precisa ter mais detalhes para implicar em formulação e implementação de políticas públicas^(15)^. Os valores em todas as etapas são considerados bastante elevados e a análise financeira da realização desses procedimentos ocorre, na maioria das vezes, sob a perspectiva de custos diretos. Para compreender o impacto financeiro real é necessário reconhecer, quantificar e valorar todos os recursos utilizados direta e indiretamente com as despesas em qualquer aquisição de órgãos^(17)^. Além disso, existem variáveis qualitativas importantes que podem impactar nessa mensuração financeira. As análises de custos aliadas aos índices de sobrevida e qualidade de vida são capazes de gerar dados importantes para os serviços públicos de saúde e cooperar com o uso eficiente de recursos que são limitados^(19)^.

Esta pesquisa observou uma correlação negativa moderada entre a lista de espera e o número total de órgãos e tecidos transplantados, mas a tendência de pacientes em espera para transplante foi crescente. Embora as terapias substitutivas atinjam resultados positivos no país, o volume de procedimentos ainda não é capaz de suprir as necessidades da população, que reflete na crescente lista de espera por órgãos, células e tecidos^(21)^.

Em 2022, a taxa de efetivação da doação foi de 26,9%, valor 20% inferior ao ano anterior. Além disso, houve um baixo índice de notificações de ME com redução de 18% neste ano. A notificação de PD foi de 13.195 pmp/ano; entretanto, o registro de DE foi de 6.423 pmp/ano com uma efetivação de doação de 3,528^(14)^. A conversão para DE ainda é insuficiente para a demanda do país com o progressivo aumento anual na lista de espera.

Neste contexto, a fila de espera por um rim assume a liderança quando comparada aos demais órgãos. O tempo médio de espera ajustado pela mortalidade é de 5,5 anos. Sem essa estimativa, o tempo é de aproximadamente 11,1 anos^(19)^, o que significa que muitas vidas são perdidas a cada ano, porque não existem órgãos suficientes para todos^(22)^.

No país, a oferta de um órgão não é inversamente proporcional à realização de transplante e muitos fatores dificultam a movimentação positiva da lista de espera, que em 2022 fechou com 52.989 pacientes^(14)^. Em um centro de tratamento por hemodiálise na região Sudeste foram encontrados 12.415 pacientes e destes 77,2% não realizaram o transplante^(4)^.

Várias são as fragilidades encontradas no SNT. Dentre vários fatores, pode-se destacar a inexistência de estratégias de gestão assistencial, em especial com o diagnóstico de ME, condução do processo de doação, habilidade em realizar manutenção do PD e a insegurança em lidar com a comunicação com a família e o processo de luto^(2)^.

Países como Espanha, Portugal e EUA têm obtido êxito em relação ao Brasil, principalmente devido ao direcionamento de seus recursos para treinar as equipes para execução de todas as etapas do processo doação-transplantação^(1)^, bem como para o aperfeiçoamento das estruturas assistenciais e investimentos em programas de qualidade que acompanham as oportunidades de progresso nas etapas deste processo^(1,2)^.

Neste estudo, a entrevista familiar correlacionou fortemente com a negativa familiar, dado que corrobora a tendência crescente do número de PD e decrescente do número DE. O diagnóstico de ME de um PD é criterioso e seguro; porém, a família assume o papel relevante na prerrogativa ética e legal de tornar este PD um DE^(22)^.

A insegurança familiar e a baixa credibilidade no sistema de saúde, aliadas a fatores culturais e religiosos, resultam na não concretização das doações de órgãos no país^(23,24)^. A ausência de um diálogo aberto e transparente em vida tem sido um obstáculo histórico para a aceitação familiar. Adiar essa decisão até a morte de um ente querido representa um desafio no aceite familiar para doação, uma vez que o processo de luto pode influenciar nessa decisão^(25)^.

O papel da família no processo da doação de órgãos e tecidos é determinante na melhoria das realidades encontradas e a não autorização familiar é o principal motivo para que um órgão não seja doado no Brasil e em vários países no exterior, com taxas que variam de 5,7 a 41,4% em países europeus, e 27,5 a 48,9% em países latino-americanos. No Brasil um estudo aponta um percentual de variação de 37,3% a 70% a depender da região^(26)^.

Dentre as prerrogativas de recusa, as de maiores índices encontradas nos estudos tratam de manter o corpo íntegro (36,0%) e a insegurança acerca do processo de doação (32,6%)^(26)^. Para que esses obstáculos sejam sanados e se eleve o número de aceites para doações de órgãos e tecidos faz-se necessário que haja um investimento prioritário, no contexto da abordagem familiar ao formular novas políticas públicas^(9)^, na capacitação dos profissionais envolvidos, e por fim, na transparência com os desfechos de todo processo disponibilizados publicamente^(23)^.

A equipe de enfermagem, assim como a equipe multiprofissional, desempenha papel fundamental na abordagem familiar e sua conduta pode ser um ponto decisivo para que haja o aceite da doação. Por meio de sua atuação, os profissionais de enfermagem podem ser um elo do serviço de saúde com os familiares ao oferecer suporte em um momento de vulnerabilidade emocional com uma comunicação sensível, escuta ativa e qualificada, apoio ao luto, oferta de orientações e esclarecimentos relacionados ao procedimento^(27)^.

Outro ponto importante trata da necessidade de a sociedade ser sensibilizada quanto à doação de órgãos. Como estratégia, o marketing social é imprescindível neste contexto, pois propicia a reflexão de sua importância, promove o diálogo familiar e pode favorecer o consentimento da doação^(26)^. A realização do transplante é de um valor incalculável para o receptor, seus familiares e também a sociedade de uma maneira geral, pois além de devolver a autonomia do indivíduo, diminui os custos dos serviços de saúde e tem potencial de devolver este indivíduo ao mercado de trabalho.

Portanto, é perceptível que os custos associados aos transplantes ultrapassam os valores disponíveis registrados nas plataformas oficiais. É essencial que se considere os fatores sociais e econômicos de indivíduo em idade produtiva, que em decorrência de sua condição de saúde está fora do mercado de trabalho impactando o setor previdenciário nacional. Além disso, as inúmeras internações, tratamentos clínicos e medicamentosos oneram o setor saúde e interferem diretamente nos recursos destinados a esta área.

Nessa perspectiva, pode-se entender que as informações apresentadas por este estudo fornecem evidências relevantes sobre o processo de doação e transplante, que é extenso, complexo e precisa ser norteado de maneira eficaz, com possibilidade de revisão de fluxos de trabalho e modelos de gestão com vistas ao melhor gerenciamento e alocação de recursos. Avaliar os resultados assistenciais e financeiros tem sido um desafio para os serviços de saúde^(2)^, mas constituem ponto vital na gestão de investimentos escassos em busca dos melhores resultados.

### Limitações

Quanto às limitações metodológicas do estudo, é importante destacar o uso de dados secundários de domínio público, o que pode introduzir um viés de informação devido à falta de controle sobre a qualidade da aferição e mensuração desses dados. Além disso, variáveis de confusão importantes como a capacidade de coordenação e logística, infraestrutura hospitalar regional, conscientização da população sobre doação de órgãos e políticas locais específicas, não foram contempladas nas fontes de informação, o que poderia ter impacto nos resultados da análise. Essas questões foram discutidas no estudo, mas sua não inclusão pode ter influenciado nos resultados de forma geral.

Além das limitações metodológicas, também é importante reconhecer outras limitações. Por exemplo, a falta de dados orçamentários relativos aos custos indiretos dos transplantes pode ter limitado a compreensão completa do impacto financeiro desses procedimentos. Além disso, a escassez de pesquisas sobre o tema limitou a base de evidências disponível para embasar as discussões do estudo.

### Contribuições Para Área da Saúde

O estudo evidencia que os custos relacionados aos procedimentos de transplantes vão além dos valores oficialmente reportados e proporciona uma valiosa contribuição para gestores em saúde pública ao sinalizar a necessidade de considerar os aspectos financeiros diretos e os fatores sociais e econômicos individuais dos pacientes em lista de espera. Além disso, por se tratar de um estudo ecológico misto que utiliza o agregado populacional das regiões brasileiras, é possível identificar de forma mais precisa quais regiões enfrentam maiores limitações nos procedimentos de transplante. Isso, por sua vez, destaca a importância de investimentos e políticas públicas mais eficazes para atender um maior número de indivíduos nessas regiões.

## CONCLUSÃO

Os números relativos aos procedimentos de transplantes realizados no Brasil contrapõem-se à lista de espera por um órgão sólido ou tecido, que ocorre em maior número nas regiões Sul e Nordeste do país. A logística de captação, transplantação e o produto interno bruto de cada região pode colaborar com o aumento destes números. Além disso, o número de negativas familiares apontadas como a maior responsável pela não doação de órgãos e tecidos está associado ao aumento da lista de espera por um órgão ou tecido.

Esta diferença entre o quantitativo de transplantes entre as regiões do Brasil reflete a necessidade de organização e novos credenciamentos das centrais de transplantes, assim como de maior divulgação de informações sobre a ME e o transplante à população, de modo a conscientizar e sensibilizar quanto à necessidade da doação de órgãos, células e tecidos.
